# A Remarkable Case of Acute Motor-Sensory Axonal Polyneuropathy (AMSAN) Variant of Guillain Barré Syndrome, in a Diabetic Patient Infected With COVID-19: A Case Report and Review of the Literature

**DOI:** 10.3389/fneur.2022.937989

**Published:** 2022-07-04

**Authors:** Sajjad Ali, Alvina Karam, Aarish Lalani, Sadia Jawed, Musfirah Moin, Zain Douba, Murtaza Ali

**Affiliations:** ^1^Department of Medicine, Ziauddin Medical University, Karachi, Pakistan; ^2^Department of Medicine, Khyber Medical College, Peshawar, Pakistan; ^3^Department of Medicine, Liaquat National Hospital and Medical College, Karachi, Pakistan; ^4^Faculty of Medicine, University of Aleppo, Aleppo, Syria; ^5^CME Office, Faculty of Medicine, University of Aleppo, Aleppo, Syria; ^6^Department of Medicine, Dr. Ruth K.M. Pfau, Civil Hospital Karachi, Karachi, Pakistan

**Keywords:** Guillain-Barré syndrome, coronavirus, COVID-19, neuroelectrophysiological characteristics, neuroinfection

## Abstract

**Background:**

Severe acute respiratory syndrome coronavirus 2 (SARS-CoV-2), which causes coronavirus disease- 2019 (COVID-19), has been a global epidemic in our healthcare system. SARS-CoV-2 primarily affects the respiratory system, but neurological involvement has also been reported, including Guillain–Barré syndrome (GBS) development.

**Case Presentation:**

A 58-year-old male with known co-morbid hypertension and type 2 diabetes mellitus presented to the emergency room with complaints of worsening shortness of breath, dry cough, and fever for the past 10 days. On day 20 of hospitalization, he developed neurological symptoms after being tested positive for COVID-19. A neuroelectrophysiology study was conducted to evaluate neurological symptoms and suggested that the patient suffers from acute motor-sensory axonal polyneuropathy (AMSAN). CSF analysis showed elevated protein levels that confirmed the diagnosis of GBS. He was subsequently treated with oral prednisolone and IVIG, which improved neurological symptoms.

**Conclusion:**

Ever since the emergence of COVID-19, GBS has surfaced as to its potentially dangerous outcome. Healthcare professionals should be mindful of GBS and should rule it out in anyone having sensory symptoms or weakness during or after a COVID-19 infection. Its early detection and treatment can result in improved clinical outcomes.

## Introduction

Severe acute respiratory syndrome coronavirus 2 (SARS-CoV-2), which causes coronavirus disease- 2019 (COVID-19), has been a global epidemic in our healthcare system with 276,939,345 cases worldwide and 5,389,206 deaths, as of December 22, 2021 (1). The most common clinical presentations of COVID-19 are fever, headache, vomiting, malaise, and respiratory symptoms, ranging from a mild cough to severe pneumonia (2). If not promptly managed, life-threatening complications such as acute respiratory distress syndrome (ARDS), multi-organ failure, and even death can occur. Moreover, due to the complexity of COVID-19, multiple systemic manifestations have also been documented (3). SARS-CoV-2 primarily affects the respiratory system, but neurological involvement has also been reported, including Guillain–Barré syndrome (GBS) (4).

GBS is a life-threatening inflammatory/autoimmune condition in which the immune system targets healthy nerve cells in the peripheral nervous system (PNS). Usually, an infectious illness triggers the condition, such as gastroenteritis or a lung infection. Progressive ascending motor impairment with reduced reflexes and cranial nerve involvement is the hallmark clinical sign of GBS, which can persist from a few days to several weeks (5). Acute inflammatory demyelinating polyneuropathy (AIDP) is the most common type of GBS in the western part of the world; acute motor axonal neuropathy (AMAN) is the most common form in the Asian region, and Miller-Fisher syndrome (MFS) is the least common form in the western world. In addition, there are overlap syndromes (GBS-MFS overlap) (6).

In light of the clinical relevance of COVID-19 during pandemic times, the authors have presented a suspected case of GBS in a COVID-19 patient in a tertiary care hospital.

## Case Presentation

A 58-year-old male with a known co-morbid of hypertension and type 2 diabetes mellitus (compliant with medications) presented to the emergency room on September 25, 2021, with complaints of worsening shortness of breath, dry cough, and intermittent fever for the past 10 days. On general physical examination, his respiratory rate was 50 breaths per minute, along with the use of accessory muscles. He had 90% oxygen saturation on high-low oxygen. With worsening respiratory symptoms, he was shifted to the Critical Care Unit (CCU).

On admission, baseline laboratory investigations were carried out (Table 1). He had low hemoglobin levels, low leucocyte count, and significantly high C-reactive protein (CRP) and Creatinine (Cr) levels. He was further tested positive for SARS-CoV-2 by polymerase chain reaction (PCR), despite being fully vaccinated for COVID-19.

**Table 1:** Baseline investigations of the patient on admission.

**Parameters**	**Result**	**Normal range**
**Complete blood count**
Hb (g/dl)	11.7	13.5–16.5
MCV (fl)	84.7	78.1–95.3
MCH (pg)	25.5	25.3–31.7
MCHC (Gm/dL)	30.2	30.3–34.4
Total leukocyte count (/L)	10,500	4,600–10,800
Lymphocytes (%)	22	17.5–45
Neutrophils (%)	67.8	34.9–76.2
Platelets count (counts/uL)	263,000	154,000–450,000
**Inflammatory markers**
CRP (mg/dL)	319.4	<10
ESR (mm/h)	76	≤ 20
**Biochemical profile**
Sodium (mEq/L)	150	135–145
Potassium (mEq/L)	3.7	3.5–5.0
Chloride (mEq/L)	115	95–105
Calcium (mEq/L)	8.9	8.5–10.2
Magnesium (mg/dl)	1.9	1.5–2.0
Phosphorous (mg/dl)	5.5	3.0–4.5
BUN (mg/dL)	39	6–24
Cr (mg/dl)	6.3	0.6–1.2
**Urine direct report**
pH	6	4.5–8
Specific gravity	1.03	1.005–1.025
Protein (mg/day)	+	≤ 150
Glucose (mmol/L)	+++	0–0.8
Blood (RBCs)	++	≤ 3
RBCs (per hpf)	20	≤ 2
Pus cells	2–3	0–4
Yeast cells	+++	none

On detailed investigation, a High-resolution Computer Tomography (HRCT) scan of the chest was done and revealed patchy bilateral areas of ground-glass opacity along with lobular septal thickening. In addition, there were bilateral extensive fibrotic patches in sub-pleural and central locations, together with pleural thickening. The above findings represent pulmonary fibrosis with interstitial lung disease due to sequelae of COVID-19 pneumonia.

The patient was intubated during the CCU hospitalization due to worsening hypoxemic respiratory failure that was refractory to noninvasive positive pressure ventilation. He was given an entire course of remdesivir and dexamethasone during the intubation period. On the 15th day of hospitalization, he was extubated and was shifted out of the CCU.

On the 20th day of hospitalization (30 days after onset of COVID-19 symptoms), the patient developed bilateral lower limb weakness. Given the concern for the neurological nature of the complaint was evaluated by an on-call neurologist. His physical exam revealed intact cranial nerves and sensations but a power of 3/5 in lower limbs bilaterally along with absent lower limb reflexes and mute plantar reflex. To rule out etiologies of polyneuropathy, a magnetic resonance imaging (MRI) scan of the cervical was done which showed to be insignificant.

On further investigation, neuroelectrophysiology (Table 2) was done whose report showed decreased compound muscle action potential (CMAP) amplitude and sensory nerve action potential (SNAP) amplitude in the left median and ulnar nerves. Moreover, electromyography (EMG) showed few motor unit action potential (MUAP) along with fibrillation in the left first dorsal interosseous and left tibialis anterior muscles. In conclusion, the reports suggested acute motor-sensory axonal polyneuropathy (AMSAN).

**Table 2:** Neuro-electrophysiology report of the patient.

**Motor nerve conduction study**									
**Nerve**	**Recording Site**	**CMAPS amplitude** **(mV)**		**Distal latency** **(m/s)**		**Proximal latency** **(m/s)**		**Conduction velocity** **(m/s)**	
		**Lt**.	**Normal value**	**Lt**.	**Normal value**	**Lt**.	**Normal value**	**Lt**.	**Normal value**
Median	APB	1.2	>4	3.2	<4.5	8.0		50.3	45–55
Ulnar	ADM	1.7	>4	2.8	<4.2	8.4		48.9	45–55
Radial	EIP		>5		1.9–2.9				55–67
Peroneal	EDB	N/R	>2	N/R	<6.5			N/R	40
Tibial	AHB	N/R	>3	N/R	<6.5	N/R		N/R	40
Peroneal	TA		>2		<3	N/R			40
**Sensory nerve conduction study**									
**Nerve**		**Latency**				**Snaps Amplitude UV**			
		**Recording value**		**Normal range**		**Recording value**		**Normal range**	
Left Median		3.0		2.9–3.5		7.3		>15	
Left Ulnar		2.8		2.9–3.5		7.0		>15	
Left Radial				1.9–2.8				>10	
Left Sural		NR		<4.2		NR		>5	
**Electromyography study**									
**Muscles**			**Insertion activity**		**Spontaneous activity**		**Motor unit potential**		
							**Amplitude (uV)**	**Duration (m/s)**	**Morphology**
Left Biceps			-		Nill		Normal	Normal	Bi,tri
Left FDI			-		Fibs+ve		Few	MUAPS	
Left Quadriceps			-		Nill		Normal	Normal	Bi,Tri
Left. TA.			-		Fibs+ve		Few	MUAPS	

*APB, Abductor pollicis brevis; ADM, abductor digiti minimi; EIP, extensor indicis proprius; EDB, extensor digitorum brevis; AHB, abductor halluces brevis; TA, tibialis anterior; FDI, (first dorsal interrossei; TA, tibialis anterior; MUAP, motor unit action potential; fibs, fibrillation*.

Moreover, a detailed cerebrospinal fluid (CSF) analysis was carried out which revealed to have isolated elevated protein levels (71 mg/dL), while the rest of the components were in the normal range such as pH, specific gravity, glucose. No white blood cells or red blood cells were noted. Culture and sensitivity, and gram staining were both negative. Increased protein levels is a hallmark of GBS.

Based on clinical findings, neurophysiological studies, CSF analysis, and MRI of the cervical, the patient was diagnosed with GBS associated with COVID-19.

Initially, the patient was given oral glucocorticoid (prednisolone) for 5 days and was tapered off when symptoms began to improve. He further received intravenous immunoglobulins (IVIG) for 6 days continuously. His vitals were stable throughout the rest of the hospital stay, and he had no signs of distress. After receiving this treatment, the patient's symptoms significantly improved, and he was discharged on October 21, 2021, to home with regular physiotherapy advised by the on-call doctor.

The present paper has been reported in accordance with the CARE guidelines (7).

## Discussion

GBS is an immune-mediated polyneuropathy that is most often preceded by infection. It has been linked with many infectious disease-causing organisms such as Campylobacter jejuni, cytomegalovirus, Epstein-Barr virus, Zika virus, as well as previous coronaviruses i.e., Middle East respiratory syndrome (MERS) and severe acute respiratory syndrome (SARS) (8, 9). In our case report, we have discussed a case of GBS in a 58-year-old male, which was caused by an aberrant immunological response following 20th days of hospitalization post-COVID-19 infection. GBS was confirmed after assessing clinical features and nerve conduction investigation revealing motor-sensory axonal polyneuropathy.

Despite being a rare GBS variant in the western world, AMSAN is more commonly associated with COVID-19 infection in Asia. We have reviewed and compiled all of the AMSAN type of GBS cases that have been associated with SARS-CoV-2, looking at their clinical presentations, the average latency period until GBS symptoms appear, the CSF findings, as well as their treatment plans and outcomes. In addition, we did a thorough literature search on the PubMed database for all studies with full text available in English and original data on GBS patients with recent COVID infection published between June 2020 and October 29, 2021. We searched using the following keywords: Guillain–Barré syndrome, GBS, COVID-19, SARS-CoV2, and AMSAN. As a result, we found a total of 11 published articles with 13 AMSAN-GBS cases associated with COVID-19 (Table 3).

**Table 3:** Review of the literature for AMSAN-GBS associated with COVID-19.

**Age, gender**	**Onset of disease**	**Clinical Features**	**CSF findings**	**Treatment**	**Outcome**
Seventy years, Female (10)	Ten days after COVID-19 symptoms	Quadriplegia, hypotonia, areflexia and bilateral positive Lasègue sign	Increased protein level at 1 g/L Normal white blood cell count	Intravenous immunoglobulin (2 g/kg for 5 days) Hydroxychloroquine (600 mg per day) and Azithromycine	No significant neurological improvement was seen after 1 week of treatment.
Sixty years, Male (11)	Day 20 of hospitalization	Acute weakness in lower limbs with distal distribution Foot drop on the right side. Gastroplegia, paralytic ileus, and loss of blood pressure control	Oligoclonal bands seen. Increased ratio IgG/albumin in CSF (170) Normal total protein level	Intravenous immunoglobulin (0.4 g/kg/day)	After 5 days, the vegetative symptomatology significantly improved, with the remission of gastroplegia and recovery of intestinal functions. Persistence of osteotendinous hyporeflexia but slight improvement in the right foot drop.
Sixty-five years, Male (12)	Two weeks after hospitalization	Acute progressive symmetric ascending quadriparesis	N.A	Intravenous Immunoglobulin (0.40 g/kg/day) for 5 days	N.A
Fifty-five years, Female (13)	Day 26 of hospitalization	Acute progressive lower limb weakness	Average glucose, cell count, and protein (57 mg/dL protein)	The patient started on intravenous immunoglobulin (20 g IV daily for 5 days)	On the third day of IVIG treatment, she developed acute respiratory distress syndrome (ARDS).
Seventy-seven years, Female (14)	Seven days after COVID-19 symptoms	Tetraplegia, areflexia, paresthesia in upper limbs, facial diplegia, dysphagia, tongue weakness, and respiratory failure.	Albuminocytological dissociation	Two cycles of intravenous immunoglobulin	Persistence of severe upper-limb weakness, dysphagia, and lower-limb paraplegia.
Twenty-three years, Male (14)	Ten days after COVID-19 symptoms	Facial diplegia, areflexia, lower limbs paresthesia, and ataxia	Albuminocytological dissociation Protein level: 123 mg/dl no cells negative PCR assay for SARS-CoV-2	Intravenous immunoglobulin received	Had improvements, including decrease in ataxia and mild decrease in facial weakness.
Fifty-seven years, Male (15)	Twelve days after the resolution of COVID-19 symptoms	Numbness and tingling in the hands and feet. Over 10 days, the patient developed distal limb weakness and severe gait impairment	Normal cell count and normal proteins Normal CSF/serum albumin ratio Absence of oligoclonal banding	Intravenous immunoglobulin cycle at 0.4 g/kg/day over 5 days	Significant improvement of the weakness in the upper limbs and the left foot but a poor benefit on the right foot and gait ataxic. Slowly improvement by physiotherapy and, after 1 month, able to walk without aid and was discharged.
Seventy-six years, Male (16)	Five to Seven days after COVID-19 symptoms	Diarrhea, coughing, and a history of 1-week common cold. Unable to move lower limbs and not able to stand or walk	High Protein: 76 mg/dL	Intravenous immunoglobulin (20 g/day for 5 days concomitant anti- COVID treatment	Discharged to home with good recovery.
Fifty-four years, Male (17)	Afebrile	Ascending progression of weakness. afebrile and severe dysautonomia	No albumino-cytological dissociation in CSF	Intravenous immunoglobulin and invasive ventilation	Passed away
Thirty-six years, Female (17)	Twelve days after COVID-19 symptoms	Ascending progression of weakness	Albuminocytological dissociation	Intravenous immunoglobulin	Discharged with good improvement.
Sixty-six years, Male (18)	One month after the COVID symptoms	Progressive ascending weakness	High protein 0.6 g/L (0.15–0.45 g/L) High glucose 3.97 mmol/L (2.2–3.9 mmol/L) Normal cell counts	Started on intravenous immunoglobulin at (0.4 g/kg IV) once daily for 5 days	Improved clinically, with power 4/5 in the upper extremities and 3/5 in of the lower extremities. The patient was discharged.
Fifty-five year, Female (19)	Twenty-six day after hospitalization	Decreased muscle strength in lower limbs	Glucose: 78 mg/dL Protein: 48,4 mg/dL (normal value: <50 mg/dL), No white blood cells (WBCs).	Intravenous immunoglobulin	Passed away due to acute respiratory distress syndrome (ARDS) before the medication could start its effect.
Sixty-one year, Male (20)	A month after COVID-19 symptoms	Flaccidity in all of the upper and lower limb muscles	N.A	Started on intravenous immunoglobulin (400 mg/kg body weight daily, for 5 days)	Significant clinical alleviation of symptoms with improvement in respiratory functions, oxygen saturation, and return of muscle power, recovering to 4/5 power in the upper and lower limbs on both sides.

*N.A, Not available; CSF, Cerebrospinal Fluid*.

A study by Toscano et al. (14) showcased five patients, three having an axonal type of GBS and two having demyelinating neuropathy. However, electrophysiological tests revealed demyelinating polyneuropathy in the majority of published cases, with axonal neuropathy in only four patients (12). Diabetes mellitus (DM) is prevalent comorbidity in some of these individuals, as it was in our case (12, 21). The clinical and electrophysiological aspects of concurrent peripheral neuropathies, including GBS, are worsened by underlying DM. The specific mechanism causing DM-induced aggravation is unknown; however, it could be linked to chronic inflammatory disorders associated with DM, as well as peripheral nerve neurovascular deterioration (22).

So far, three cases have been documented in the literature, two with sphincter dysfunction and one with hypertension (23). However, there were no symptoms suggestive of autonomic system disturbance in our patient or the five cases reported from Italy (9). In addition to the conventional post-infectious pattern, numerous instances of GBS post-COVID-19 had followed a para-infectious course (24).

Various case reports and series have been published, suggesting a possible association between SARS-CoV-2 infection and GBS (2), although the actual mechanism is still unknown. According to one explanation, nerve tissue injury is linked to the virus's direct neuronal invasion by directly binding to ACE2 receptors. Another theory explains immune-mediated indirect neuron injury by claiming that the immune system is overstimulated, leading to increased interleukin-6 (IL-6) production and the emergence of an autoimmune reaction (25). SARS-CoV-2 has been shown to promote an overactive immunological response, activate inflammatory cells, and enhance the production of cytokines such as IL6, resulting in neural tissue damage. As a result, it is possible that these immunological processes are to blame for COVID-19 individuals' neurological symptoms. COVID19 patients with severe symptoms and a rapidly deteriorating clinical status are also more likely to experience serious neurological events, according to the research. It is currently unclear if COVID-19 causes the production of autoantibodies against specific gangliosides, as found in other GBS types. In the future, more research into the pathophysiologic mechanism of GBS in COVID-19 patients will be needed.

## Conclusion

Healthcare professionals should be conscious of GBS, an uncommon complication related to COVID-19. In asymptomatic patients or those who have had a mild respiratory ailment weeks earlier, diagnosis might be challenging and time-consuming. GBS should be ruled out in anyone with sensory symptoms or weakness during or after a COVID-19 infection. Clinical outcomes can be improved by early detection and treatment.

## Data Availability Statement

The original contributions presented in the study are included in the article/supplementary material, further inquiries can be directed to the corresponding author.

## Ethics Statement

Written informed consent was obtained from the individual(s) for the publication of any potentially identifiable images or data included in this article.

## Author Contributions

SA conceived, designed, supervised the study, responsible for data collection, and literature review. AK, AL, and MM analyzed and/or interpreted the data. AK, AL, SJ, and MM wrote the initial manuscript. ZD critically revised the manuscript. MA provided detailed insights of the patient's history and supervised the study. All authors have read the final manuscript.

## Conflict of Interest

The authors declare that the research was conducted in the absence of any commercial or financial relationships that could be construed as a potential conflict of interest.

## Publisher's Note

All claims expressed in this article are solely those of the authors and do not necessarily represent those of their affiliated organizations, or those of the publisher, the editors and the reviewers. Any product that may be evaluated in this article, or claim that may be made by its manufacturer, is not guaranteed or endorsed by the publisher.
